# A Dead Journalist

**Published:** 1890-05-03

**Authors:** 


### The Doctor's Pulpit.

### A DEAD JOURNALIST.

Some men write because they have something to say; others, because they have to say something. The pure journalist by no means always lives to write, though he must always write to live. The great world knows very little about journalists, living or dead. The trade is new and has hardly yet got itself shaken down into its natural and permanent resting place. Fathers with marriageable daughters, and shopkeepers who give credit are a little uncertain of their ground when a journalist is in question. He may be a person of the most eminent respectability, whose university degree is unchallengeable, whose nails and shirt fronts are alike without reproach, and whose credit is as solid as the Bass Rock at the mouth of the Firth of Forth. Or he may be the opposite of all these things; without status, or connections, or credit, or clean nails, or even a permanent "den" to hide himself in. Small wonder, therefore, that the British parent and the British shopkeeper both assume towards him an attitude of caution, and of polite, though very careful enquiry as to antecedents.

Quite recently Mr. Robertson Nicholl, himself a journalist of reputation, wrote the life of Mr. James Macdonell, also a journalist. Very few such "lives," so far as we know, are available for the enlightenment of the reading public. Yet journalism and journalists play a most important part in every department of the life of to-day. Politics and pen making, religion and red sandstone, law and laundry work alike have their journals, their editors, their leader writers, their news collectors, their occasional contributors, their printers' devils, and all the rest of it. Moreover, there is no doubt that the printed paper exercises a powerful influence over the minds of even the most self-willed and self-reliant of men. When it is considered what tens of thousands of journalists there are in this country, and how various is their culture and no-culture, it is absolutely wonderful how small is the quantity of downright rubbish that is given to the world week by week. Some people affect a lofty contempt for newspapers. That is not philosophical. If the papers do nothing else they show, at least, what is the state of the political, religious, and intellectual weather at any given time. Speaking generally, it may be said that he who does not know when it is summer and when winter, when the cold pinches and when a deluge of rain is pouring down, has a very unserviceable nervous system. On the other hand, we cannot help expressing the conviction that it will be a bad day for everybody when people universally acquire their information and take their opinions solely from newspapers. Original thought is the parent of development and the salvation of the mind.

Mr. Macdonell commenced writing before he was twenty and died before he was forty. He began his career on the *Aberdeen Free Press* and closed it on the *Times*. In the interval he had worked on the *Edinburgh Daily Review*, a semi-religious daily; on the *Newcastle Express*, a paper of radical politics; on the *Daily Telegraph*, whose politics are inscrutable; and finally on the *Times*. By the proprietors of each paper he was held to be a brilliant and successful writer and an excellent man. He wrote also for the monthly magazines and the leading reviews. He was, in short, a typical journalist of the brightest, cleverest, wholesomest, and most successful kind.

What then did this man do for his profession, what for the world, and what for himself? He played the part of a literary filter, which both clears and colours that which is passed through it: that was one thing: and he killed himself by his work before he was forty, that was another. Most of the proprietors of the newspapers for which he worked, were, at the same time, living comfortable and healthy lives, and some of them were growing enormously rich. That is worth thinking about from the standpoint both of journalists and of readers. Mr. Macdonell's intellect and character were of a certain value to himself and to his family. They also had a value for reading men and for the world. Regarding them as capital, the press-man may ask himself the question whether the capital yielded an adequate rate of interest to its owner; regarding them as serviceable endowments, the general reader may ask whether they rendered to the world the most and the best service they were capable of rendering. Mr. Macdonell's brains and character were practically his only capital. He put them out to interest, and by his management of them succeeded in getting rid of both principal and interest in something less than twenty years. We do not now ask whether he acted rationally, and so showed one of the very highest characteristics of intellect, in thus spending his life. It would probably be said by his biographer and his friends that family necessities thrust him early into journalism, and kept him there until a re-arrangement of his life-work was no longer possible. But we ask whether his experience is at all common among journalists; whether journalists contemplate such an experience with disapprobation or with sheer helplessness; whether young men who are proposing to take up the journalistic profession are sufficiently enlightened as to the kind of outlook that lies before them? Does Mr. Macdonell's life act as a stimulus or as a warning? We do not hesitate to say that it ought to act as a warning. Considering the mental capacity of Mr. Macdonell, and his character, his life must be held to have been a failure. It was certainly a failure if we compare in quality and quantity what he had it in him to do with what he actually performed. For one thing his personality was absolutely lost and wasted. He was not even a voice. For none but the very few initiated knew when they were and when they were not reading his articles.

Journalism, to the journalist pure and simple, is probably almost always a failure. It is entirely a bread winning trade like shoe-blacking or anything else. Unlike the preacher. the individual journalist seldom or never emerges at all. But the man of the first journalistic capacity is one whose individuality ought to emerge. The journalist himself fails to put forth his full powers, and to exercise his due influence when his personality is unknown; and the great world is more the loser by the want of that influence than even the journalist. In this world great personalities are much more potential than great words. Journalists who prefer to write anonymously commit a kind of intellectual and moral suicide.

Had Macdonell always written over his own name, he might have been not only now alive, but a wholesome and illuminating factor in the State. Mr. Robertson Nicholl, in writing almost the first life of a journalist that has ever been written, might with advantage have discussed the journalist's profession in all its most important aspects. In failing to do this he has failed to render to an important body of men a great and lasting service which he might have rendered. Perhaps the next hero who dies comparatively unknown in the thick of the intellectual and moral conflict of our times may find a biographer who will use his story for the enlightenment and better guidance of all his fellow soldiers.

